# Constant pH Simulation with FMM Electrostatics in GROMACS. (B) GPU Accelerated Hamiltonian Interpolation

**DOI:** 10.1021/acs.jctc.4c01319

**Published:** 2025-02-07

**Authors:** Bartosz Kohnke, Eliane Briand, Carsten Kutzner, Helmut Grubmüller

**Affiliations:** Theoretical and Computational Biophysics, Max Planck Institute for Multidisciplinary Sciences, Am Fassberg 11, 37077 Göttingen, Germany

## Abstract

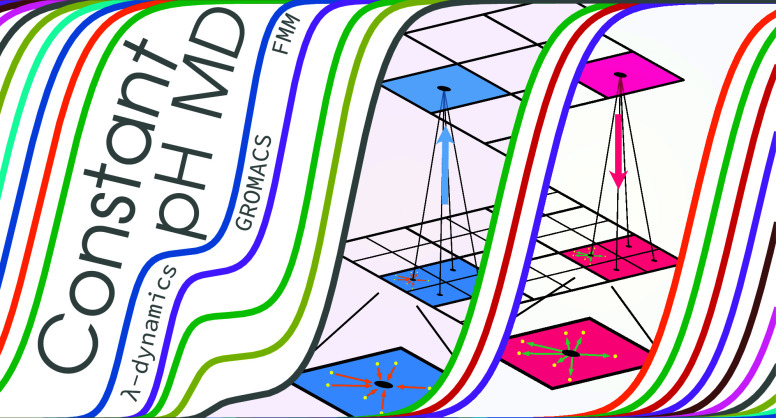

The structural dynamics of biological macromolecules, such as proteins, DNA/RNA, or their complexes, are strongly influenced by protonation changes of their typically many titratable groups, which explains their pH sensitivity. Conversely, conformational and environmental changes in the biomolecule affect the protonation state of these groups. With a few exceptions, conventional force field-based molecular dynamics (MD) simulations do not account for these effects, nor do they allow for coupling to a pH buffer. The λ-dynamics method implements this coupling and thus allows for MD simulations at constant pH. It uses separate Hamiltonians for the protonated and deprotonated states of each titratable group, with a dynamic λ variable that continuously interpolates between them. However, rigorous implementations of Hamiltonian Interpolation (HI) λ-dynamics are prohibitively slow for typical numbers of sites when used with particle mesh Ewald (PME). To circumvent this problem, it has recently been proposed to interpolate the charges (QI) instead of the Hamiltonians. Here, in the second of two companion papers, we propose a rigorous yet efficient Multipole-Accelerated Hamiltonian Interpolation (MAHI) method to perform λ-dynamics in GROMACS. Starting from a charge-scaled Hamiltonian, precomputed with the Fast Multipole Method (FMM), the correct HI forces are calculated with negligible computational overhead. However, other electrostatic solvers, such as PME, can also be used for the precomputation. We compare Hamiltonian interpolation with charge interpolation and show that HI leads to more frequent transitions between protonation states, resulting in better sampling and accuracy. Our accuracy and performance benchmarks show that introducing, e.g., 512 titratable sites to a one million atom MD system increases runtime by less than 20% compared to a regular FMM-based simulation. We have integrated the scheme into our GPU-accelerated FMM code for the simulation software GROMACS, allowing easy and effortless transitions from standard force field simulations to constant pH simulations.

## 1 Introduction

The pH of a solution is of vital importance to biomolecules, as evidenced by its tight regulation in the cellular environment. Even small deviations of 0.6 pH points from physiological values can be incompatible with life,^1,2^ as pH controls the structural integrity of proteins^3,4^ and affects important catalytic processes.^4−6^ For a more accurate description of biomolecules by molecular dynamics (MD) simulations, proper control of pH is therefore vital. Analogous to controlling temperature *T* and pressure *P* by a thermostat and a barostat,^7−9^ respectively, controlling the pH would allow a dynamically changing protonation state while—ideally—producing the same average protonation and fluctuations as under experimental conditions.

Unfortunately, with a few exceptions,^10−13^ a computationally simple yet accurate “acidostat” for the protonation chemical potential  is not a common feature of MD simulation packages. Although over the past years a number of such techniques have been proposed, among those discrete switching of the protonation state based on intermittent Monte Carlo moves,^13−18^ continuous switching,^12,19−21^ and various flavors of λ-dynamics,^10,11,22−30^ all collectively referred as constant pH MD. These techniques often require extensive enhancements of the underlying simulation code,^30^ typically at a significant cost in computational speed and increased simulation protocol complexity.^28,31^ Among these techniques, λ-dynamics has emerged as the preferred approach for explicit solvent constant pH MD simulations.

Similarly to free energy perturbation (FEP)^32^ or thermodynamic integration (TI),^33^ λ-dynamics describes a system of interest using sub-Hamiltonians for different protonation states. For example, in the simplest case of a single titratable molecule, two sub-Hamiltonians are used to represent the protonated state  and the deprotonated state . Their combination yields the full Hamiltonian
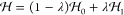
1using a continuous variable λ to linearly interpolate between both possible end states. This approach is referred to as Hamiltonian interpolation (HI). Unlike both TI and FEP, where λ is a control parameter, λ-dynamics associates λ with a mass *m* and a velocity λ̇, making λ a “pseudoparticle” whose time evolution is governed by an extended Hamiltonian
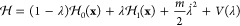
2on par with the Cartesian coordinates **x** of all “real” particles of the system. The term *V*(λ) is essential to achieve a sufficiently accurate description and control of the protonation thermodynamics and kinetics, as explained in detail in our companion publication.^34^ Here we will focus on aspects specific to electrostatics, in particular on the efficient calculation of the pseudoforce on the λ particle
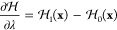
3which is required to calculate λ-dynamics trajectories. In the following we will continue using the  notation for simplicity, bearing in mind that it contains only the electrostatics terms.

The calculation of long-range electrostatic forces is a notorious challenge and efficiency bottleneck in modern MD simulations.^35−37^ These forces decrease as 1/*r*^2^ and, due to their long-range nature, must be computed across the entire simulation box, leading to an  calculation scheme for *N* particles, which would severely limit simulation system size without more efficient approximation methods. The de facto standard electrostatic solver for MD simulations, Particle-Mesh Ewald (PME),^38^ relies on Fast Fourier Transforms (FFT) to calculate the long-range part of the electrostatic interactions of periodic systems using discretized grid-based charges.

For rigorous HI with PME, the evaluation of  (eq 3) requires separate computations for the two sub-Hamiltonians  and , where each sub-Hamiltonian requires a separate grid and thus a separate FFT, which is the most communication-intensive and therefore performance-limiting part of the parallel PME algorithm.

For systems with many titratable sites, PME-based HI results in a computational effort scaling linearly with the number of Hamiltonians, which would render it impractical for constant pH simulations with larger number of titratable groups. To overcome this problem, alternative methods such as charge interpolation (QI) (aka charge scaling) have been proposed,^10,11,26^ where, instead of the Hamiltonians, the partial charges are interpolated, which allows for efficient computation of the force on the λ particle while still using PME. HI and QI generally produce different forces on λ particles, but the implications of these differences remain poorly understood. Although both methods have been widely used, further investigation has been limited by the high computational cost of HI, mainly due to a large overhead of additional long-range electrostatics calculations.

Despite the prevalence of FFT-based methods for long-range electrostatics in MD, alternatives such as the Fast Multipole Method^39^ (FMM) exist, which scales asymptotically linearly with respect to the number of particles. The method approximates the potential and the forces with a hierarchical scheme of multipole-multipole interactions. Due to the hierarchical decomposition of the simulation volume, good parallel scalability is achieved,^40^ too, as the communication effort scales as  with the number of computational nodes *P* in contrast to PME, where  requirement limits parallel scaling.^37^ In addition, the spatial decomposition of the computational domain and the ability to separate the periodic and nonperiodic parts of the calculation open up new possibilities for MD simulations, such as sparse systems like aerosols or droplets,^41^ systems with open boundaries,^42^ and, most importantly for this work, Hamiltonian interpolation for λ-dynamics.

Here we develop and assess an efficient implementation of HI-based λ-dynamics for systems with a large number of titratable sites. We introduce a scheme that allows an efficient calculation of larger numbers of  values. As a result, our implementation requires almost no additional computational effort even for large numbers of protonatable sites and, hence, λ particles. While constant pH simulations are a natural application for our method, FMM-based HI has the potential to go beyond this by exploiting the flexibility of both FMM and HI. This combination allows to tackle more complex scenarios with multiple Hamiltonians that differ not only in a few charges but also, e.g., in the number of atoms. We have integrated this scheme into our GPU-accelerated FMM code^42,43^ for the simulation software GROMACS.^37,44^ As a result, as described and tested in detail in our companion publication,^34^ HI for constant pH MD of large protein simulation systems and long time scales has the potential to be used in a straightforward way, with the simulation setup effort similar to established fixed-protonation simulations and at small runtime overhead. To demonstrate the practical consequences of choosing HI over QI, we have identified some of the differences between them.

## 2 Theory

The FMM implementation used for the present work, as well as its optimizations and accuracy/performance evaluation have been reported previously.^42,43^ After a brief summary of the FMM, here we describe the FMM extensions relevant for λ-dynamics.

### 2.1 Fast Multipole Method

**Figure 1:**
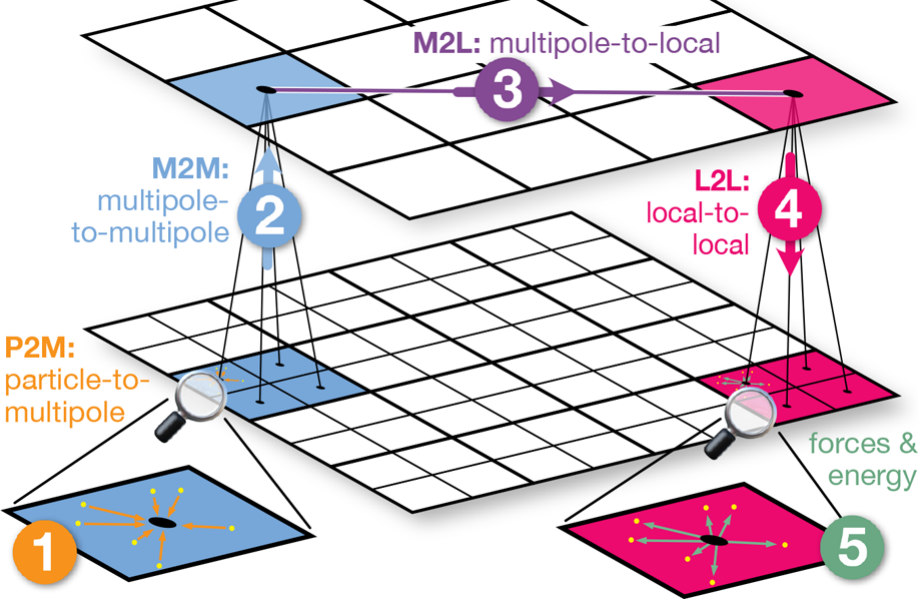
FMM far field calculation. The five individual steps and operators involved in the far field calculation, shown for the lowest two levels of the octree: ① P2M: at the lowest level, the individual charges (yellow dots) are combined into a multipole representation. ② M2M: the multipoles of the higher levels are derived from those of the lower levels (blue). ③ M2L: the multipoles (blue) are transformed into local moments (pink) at each level of the tree. ④ L2L: the local moments are propagated down the tree to the deepest level. ⑤ The local moments are used to calculate the far field contribution to the forces on the particles.

FMM approximates electrostatic interactions between *N* particles by grouping them into a near field and a far field based on their mutual distances (see Figure 1). In the near field, particle–particle interactions (in short P2P) are directly evaluated via the Coulomb sum, whereas the far field interactions are approximated as multipole–multipole interactions, truncated at a prespecified multipole order *p*.

To group interactions into near and far field, the cubic simulation box is hierarchically divided into eight equally sized sub-boxes, resulting in an octree. At depth *d*, all particle–particle interactions between adjacent boxes are considered to be near field, whereas interactions between distant boxes are assigned to the far field. The depth *d* of the octree is selected based on the system particle count *N*, ensuring a balance between near and far field computational effort to optimize performance.

The current version of our FMM-based constant pH implementation is limited to cubic simulation systems. Future updates will allow for noncubic shapes, providing greater flexibility for a wider range of molecular systems.

### 2.2 Hamiltonian Interpolation Formulation

Throughout this manuscript, we will use the term site for all atoms of a titratable group that change their partial charge upon protonation/deprotonation. Accordingly, we will use the term form to refer to a chemically distinct state of each titratable site. E.g., the simplest site comprises a protonated and a deprotonated form, where each form is described by a different Hamiltonian such as in eq 1. Generalizing this approach, a system that contains two titratable sites can be described by recursively expanding the Hamiltonian (eq 1)
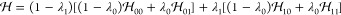
4where , , describes the electrostatic interactions
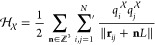
5where both sites are protonated (00), one of two sites is protonated (01 and 10), and both sites are deprotonated (11). Here, **n** is a box shift vector, which describes the periodicity of the system, **r**_*ij*_ is a vector difference between positions **x**_*i*_ and **x**_*j*_, *L* the length of the cubic simulation box, and ,  are the partial charges of particles *i* and *j* according to their form and site. The prime at the sum symbol indicates that self-interactions, i.e. interactions between particles at positions **x**_*i*_ and **x**_*j*_, where *i* = *j* and **n** = 0, are omitted. The recursive formulation, exemplified by eq 4 for two sites, is generalized straightforwardly to systems with *M* sites. Note, however, that its naive implementation would require a separate evaluation of all 2^*M*^ sub-Hamiltonians  that contain all pairwise combinations of all different forms of all sites.^28^ This approach incurs a significant computational overhead,^45^ and quickly becomes impractical for systems with many sites.

**Figure 2:**
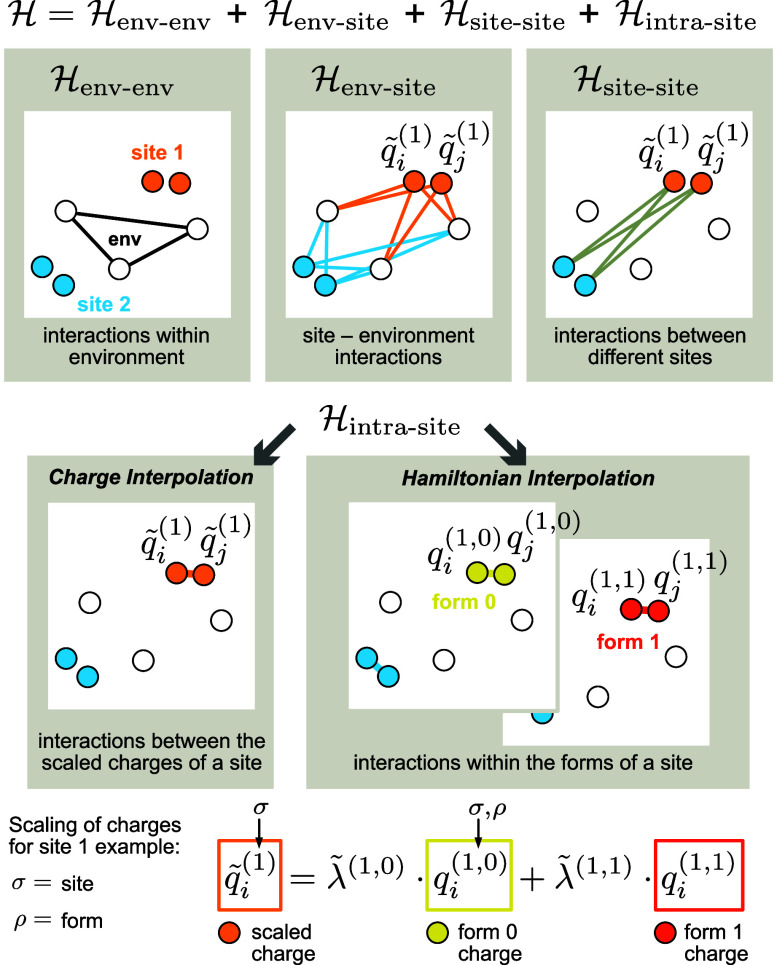
Sketch of the four different types of interactions that occur in an MD system with titratable sites. Each gray box illustrates one term of eqs 6 and 12, with particles as circles and interactions as lines. The first three terms (top three boxes) are calculated from scaled charges (*q̃*_*i*_, orange circles) and are identical for Hamiltonian (HI) and charge interpolation (QI). HI differs from QI for the intra-site interactions (gray boxes in the middle), which are calculated from scaled charges for QI (left), but from pure charges (yellow and red) for HI (right). Scaled charges are obtained by weighing form 0 (yellow) and form 1 (red), as seen at the bottom.

To overcome this limitation, we switch to a mathematically equivalent formulation for describing the Hamiltonians in λ-dynamics.^46^ In general formulation, we consider systems with *M* sites, where each site *S*^(σ)^, σ = 1,..., *M* contains *N*^(σ)^ particles that change their partial charge upon protonation. To allow for any number of forms *#S*^(σ)^ per site *S*^(σ)^, we extended the three-state model^31^ to a multistate model, where each site *S*^(σ)^ contains *N*^(σ)^ differently charged particles according to its form *S*^(σ,ρ)^, ρ = 0,..., *#S*^(σ)^ – 1. For clarity of notation here and subsequently, we will omit the interactions between periodic images. The general formulation is given by
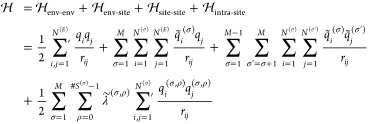
6where *N*^(*E*)^ is the number of nontitratable particles, e.g. water molecules, ions or parts of the protein not affected by protonation, and
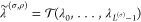
7are obtained by the transformation , where *L*^(σ)^ := ⌈log_2_(*#S*^(σ)^)⌉, with ⌈·⌉ represents the ceiling function, to round up the number of λ values to the next integer.  transforms the original λ values, as used in eq 4, to λ̃ values that describe the degree to which each form of a site (site-form) is present in the system. By construction, each λ̃ has a value between zero and one, and
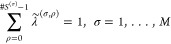
8The transformation is described in detail in the Appendix. For constant pH simulations, the original λ values reflect the progress on the protonation reaction coordinate, whereas the λ̃ weights describe the concentration of protonated or deprotonated species produced by the protonation reaction, normalized to the unit interval [0, 1]. Accordingly, the charges
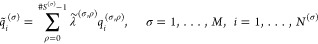
9are λ̃ scaled charges. In eq 6 the interactions are decomposed into four different types (see also Figure 2):

1. contains all interactions for which none of the atoms associated with the charges *q*_*i*_ and *q*_*j*_ are part of any site. We will call the λ-independent part of the system *environment*. These interactions do not contribute to .
2. contains interactions between the environment and atoms that are part of a titratable site.
3. contains interactions between atoms of different sites.
4. contains interactions between particles that belong to the same titratable site in a given form.

### 2.3 Multipole-Accelerated Hamiltonian Interpolation (MAHI)

MD simulations of solvated biomolecules typically employ periodic boundary conditions to avoid boundary artifacts. In addition, Ewald methods naturally yield intrinsic system energies, which are characterized by tinfoil boundary conditions at infinity.^47^ To ensure consistency with Ewald methods and to rigorously describe the specifics of MAHI, according to the computational structure of the FMM for systems with periodic boundary conditions,^48^ the Hamiltonian is split into three parts
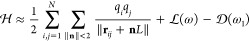
10

These parts are (i) the box-box interactions of the near field and the far field, (ii) the periodic lattice contribution , and (iii) the dipole compensation . Here, ω is the multipole of the simulation box and ω_1_ is the dipole. The lattice far field operator  approximates the periodic interactions between the simulation box and its infinite number of copies. The dipole compensation is a function of the dipole ω_1_ of the simulation box and it ensures that the tinfoil boundary conditions at infinity are met, so that the energies match those obtained by Ewald methods. Both terms  and  as well as their derivation are described elsewhere.^47,48^

MAHI efficiently retrieves energy differences, and thus forces on the λ particle, by correcting the precalculated charge-scaled Hamiltonian. While HI excludes certain intra-site interactions, QI calculates the charge-scaled interactions identically for all particles, regardless of their site-form affiliation. This allows for optimal grouping of particles into multipoles based on their spatial distribution, which is essential for FMM to achieve optimal performance. Since the corrections apply to only a few particles in the system, direct  calculations are much faster than reapplying the FMM far-field operators. Additionally, FMM enables efficient corrections of periodic interactions.

To describe MAHI, we consider a system with multiple sites with particles that can vary in their partial charge according to their form. First, the charge-scaled Hamiltonian
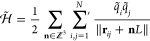
11for *N* = *N*^(*E*)^ + *N*^(1)^ + ··· + *N*^(*M*)^ particles is calculated. Since this calculation is performed on scaled charges *q̃*_*i*_, the grouping into *M* sites does not affect this stage of the calculation. Consequently, the calculation is as efficient as in a fixed-protonation simulation with the same number of particles *N*. Note that the precomputation of *N*^(1)^ + ··· + *N*^(*M*)^ scaled charges *q̃*_*i*_ is negligible in performance, and for all environment particles *q̃*_*i*_ = *q*_*i*_ holds.

To describe the next step of MAHI, we consider the difference between  and . To this end, the charge-scaled Hamiltonian is rewritten to emphasize the grouping into *M* sites according to the general formulation (eq 6), which yields
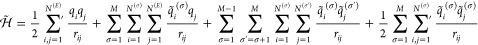
12This differs from the general formulation only in the last term (see also Figure 2), which describes intra-site interactions, i.e., interactions between particles of the same form within a site. Hence, to calculate the forces , only the intra-site interactions of the charge-scaled Hamiltonian  need to be modified. For this purpose, the corrections
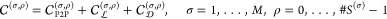
13are applied, which, according to eq 10, separately target (i) the box-box interactions (near field and far field), (ii) the lattice interactions, and (iii) the dipole compensation. The corrections for each form are then subtracted from the charge-scaled potential, weighted by the corresponding partial charge present in a given form; the procedure is described in detail in the Appendix. In the following each of the three different correction steps will be described in detail.

For compact notation, the correction charges for each form *S*^(σ,ρ)^ of *M* sites *S*^σ^ are abbreviated by
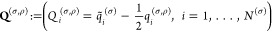
14Note that the factor 1/2 is necessary to obtain the correctly scaled  energies. The derivation of the correction term is provided in detail in the Appendix. The dipole compensation correction charges are abbreviated by
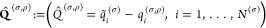
15The multipole expanded at the center of the simulation box with charges
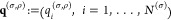
16is defined as
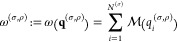
17where  is an operator calculating the multipole expansion of the simulation box.^43^ The local expansion of the simulation cell is obtained via the lattice operator
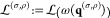
18

#### 2.3.1 Box–Box Interactions Correction

The box–box correction terms are calculated for each site-form as
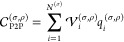
19where
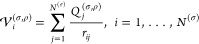
20is a correction potential evaluated between atoms within a site (i.e., intra-site). Since the number of particles *N*^(σ)^ per site is typically small, the calculation of the correction potential  has only a negligible computational overhead and can therefore be computed directly by evaluating the particle–particle interactions. Note that the interactions are calculated independently for each site-form, which leads to a straightforward parallelization of this correction part.

#### 2.3.2 Lattice Correction

The correction of the lattice part is calculated as
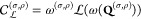
21The computational costs of ω^(σ,ρ)^ and ω(**Q**^(σ,ρ)^) operations are of the order of , so they are negligible in runtime, since *N*^(σ)^ is typically small (5–15 particles). The  lattice operator , which is the most computationally expensive part of the far field evaluation, is also negligible in runtime even though it must be computed for each site-form. This is because the total number of site-forms in a typical constant pH MD system, and therefore the number of applications of the correction lattice operator, is expected to be a small fraction of all far field operators used by FMM.^43^ Notably, similar to the box-box interactions correction , all lattice operations for different site-forms are independent and are readily parallelized using the existing unmodified CUDA lattice operator kernels.

#### 2.3.3 Dipole Compensation Correction

The dipole compensation  (eq 10), which contributes to the total energy of a system, is evaluated as described elsewhere.^47^ The correction for the dipole compensation
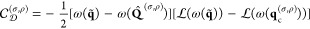
22must also be applied. To perform this operation, it is necessary to evaluate all multipoles ω(**Q̂**^(σ,ρ)^) and ω(**q**_c_^(σ,ρ)^) for each site-form. The evaluation of ω(**Q̂**^(σ,ρ)^) depends on *N*^(σ)^ and is therefore insignificant in runtime. The evaluation of ω(**q**_c_^(σ,ρ)^) is performed on fictitious charges for the dipole correction (**q**_c_^(σ,ρ)^), which are placed at the corners of the simulation box for the purpose of calculating the dipole compensation.^47^ There are four charges used for calculating the dipole-compensating multipole and 46 charges used to calculate the dipole-compensating local moment. The calculation of these charges is negligible in runtime. Although the correction requires an evaluation of the charge-scaled multipole ω(**q̃**), this calculation is required only once for the whole system, so performance is not affected. The  lattice operation  is performed for each site-form, rendering the computational overhead identical to that of the lattice correction. Another lattice operation  is evaluated only once for the whole system because it depends on ω(**q̃**). When the dipole compensation is engaged, the additional term  has to be added to the lattice term  resulting in an updated expression
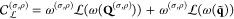
23This correction leverages also the precomputed term , rendering the total computational costs negligible.

#### 2.3.4 Execution of MAHI

**Figure 3:**
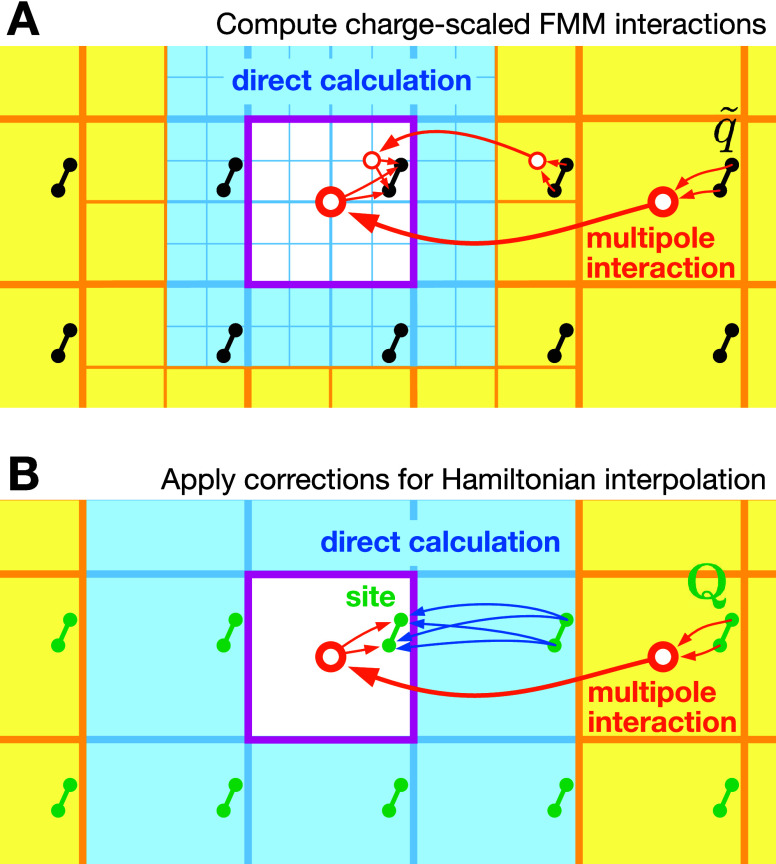
Starting from a charge-scaled Hamiltonian, the MAHI scheme calculates the correct (periodic)  interactions for Hamiltonian interpolation (HI). The central magenta box shows the actual simulation volume containing a two-atomic site (black/green dots), while the surrounding boxes are periodic images. (A) First, FMM calculates the interactions for the scaled charges *q̃* using multipole expansion in the yellow areas. (B) Corrections are then computed so that HI is retrieved for a site with charges **Q**_P2P_ and  (green). Here, in contrast to a regular FMM, all corrections to interactions coming from the first layer around the central box are computed directly (blue), while corrections from distant boxes are handled by a lattice operator (yellow).

Figure 3 illustrates how the corrections are applied. The FMM computes interactions between particles in the same box and between neighboring boxes at the deepest level *d* of the octree directly, while the remaining interactions are evaluated via far field operators. In contrast, the  part of MAHI calculates all interactions between particles in the central simulation box and their corresponding first periodic images as direct interactions. This is equivalent to a FMM run at tree depth *d* = 0, and it maximizes the performance of MAHI by avoiding unnecessary use of  operators for typically few particles of a titratable site. Only the more distant periodic images are corrected with the corresponding lattice operators.

#### 2.3.5 Calculation of Forces on λ Particles

To compute the forces on the original λ particles using the correction terms , an additional step is required to map the forces  to . In general, this mapping uses index tuples obtained from the transformation  (eq 7, described in detail in the Appendix). The forces are transformed according to
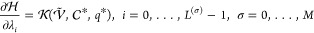
24where  is the charge-scaled potential,  is a list of all correction terms, and q* represents the site-form particles present in the system. The mapping  is described in detail in the Appendix.

#### 2.3.6 Complexity Evaluation of MAHI

To evaluate the computational complexity of MAHI, consider a system of *N* particles. The subdivision of the system into *M* sites *S*^(σ)^, where σ = 1,...,*M*, does not increase the total number of particles. Thus, the computational complexity of the electrostatics solver used for the precalculation of  does not depend on the subdivision *N* = *N*^(*E*)^ + *N*^(1)^, ..., *N*^(*M*)^, and therefore remains constant with respect to the growing number of sites. The number of applied corrections , however, depends on the number of site-forms . Since both the *N*^(σ)^-dependent part , as well as the *N*^(σ)^-independent parts  and , require only a constant amount of work independent of all other site-forms, the overall complexity of MAHI is .

### 2.4 Comparison of Hamiltonian and Charge Interpolation

Before presenting the accuracy and performance benchmarks for our MAHI, we highlight the main differences between the Hamiltonian Interpolation (HI) implemented here and charge interpolation (QI). Both methods have been successfully used for λ-dynamics simulations. While early work tended to focus on HI, recent λ-dynamics implementations have turned to QI as it can be efficiently implemented using the PME electrostatic solver.^11,29^ As can be seen from comparing the QI Hamiltonian  (eq 12) with the HI Hamiltonian (eq 6), the respective forces  and  differ only by intra-site interactions ( in Figure 2, see Section 2.3). Typically, these involve chemically directly bonded atoms, or atoms bonded to a common neighbor, and are therefore excluded from the calculation.^49^ Other intra-site interactions are not excluded, however, and therefore do matter, such as the 1–4 interactions between the proton and the other oxygen in the carboxyl group of aspartic acid (Asp) or glutamic acid (Glu). Also interactions between the proton and the other nitrogen in the imidazole moiety in histidine (His), or the backbone oxygen and nitrogen with the side chain heteroatoms, are typically not excluded and therefore, too, contribute to differences between HI and QI. Further differences are caused by interactions between otherwise excluded atoms and their periodic images.

To explore the differences resulting from intra-site interactions in more detail, consider a single site with two forms differing in *n* partial charges. Again omitting interactions between periodic images for brevity of notation, the correction term simplifies to
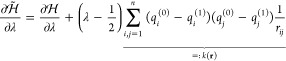
25where *r*_*ij*_ is the distance between atoms *i* and *j* of the site. As the charges of the end states , , , and  do not change, the factor *k*(**r**) depends only on the atomic positions. Integrating over λ yields
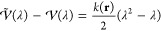
26as a λ dependent potential difference between QI and HI. This difference is a harmonic potential centered at λ = 0.5, with a force constant *k*(**r**), which is present in QI, but absent in HI. This barrier or well vanishes only at exactly λ = 0 and λ = 1. However, “protonated” or “deprotonated” states correspond to ensembles of states around these values of λ. Consequently, the QI and HI Hamiltonians and their corresponding free energies do differ. Therefore, we expect HI and QI to behave differently. Due to the calibration fit used in λ-dynamics, whose purpose and method is detailed in the companion manuscript, the effect of the additional potential is not directly deducible from the sign of *k*(**r**). Therefore, we have empirically investigated the difference between HI and QI, all else being equal, in Section 4.2. We will see that this difference affects the protonation/deprotonation kinetics and thus the convergence of constant pH simulations.

## 3 Accuracy and Performance Assessment

We have shown earlier^42^ that the electrostatic potential and the forces calculated using our FMM implementation within the GROMACS suite for nonperiodic boundary conditions approach the analytical solution with increasing multipole order *p*. For periodic boundary conditions (PBC), FMM with multipole order *p* = 8 achieves the same single precision accuracy for both energies and forces as PME with standard parameters for typical MD systems. We have also shown that for multipole order *p* = 50 our FMM implementation yields the analytic solution for a periodic lattice system within double precision accuracy.

To demonstrate that also our MAHI yields correct forces on the λ particles in PBC settings, we compared  with reference values obtained from regular FMM (or PME) electrostatics for individual Hamiltonians (i.e., for λ_*i*_ = 0 and λ_*i*_ = 1). Both individual Hamiltonians were then used to obtain total energies for arbitrary intermediate λ values to check the accuracy of our method between these end states.

Furthermore, we examined how the computational performance of MAHI scales with the number of titratable sites and forms. For constant pH simulations under physiological conditions (i.e., pH ≈ 7), most sites comprise either two (Asp, Glu) or three (His) forms. To also assess computational performance for future generalized applications, we considered up to 16 forms per site.

### 3.1 Description of the Benchmark Systems

Here we briefly describe the benchmark systems that were used to evaluate the accuracy and performance of our implementation.

#### 3.1.1 Random Systems

Random systems comprised 1000 environment particles and 10 site particles with charges drawn from a uniform distribution between −1 and +1. Two systems were constructed to evaluate a typical case and a hypothetical worst-case. For the typical case, the site particles were positioned in close proximity to each other, mimicking typical biological systems where particles of the same site usually belong to a single amino acid. For the worst case, site particles were uniformly distributed within the entire simulation box. While quite unrealistic, the latter system provides lower bound for simulation accuracy and efficiency. The same systems were used for tests with two and four forms.

To assess the accuracy of MAHI, the forces on the λ particles were compared with reference forces. The latter were obtained by computing required Hamiltonians  with a separate FMM run and using
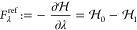
27for the system with two forms and
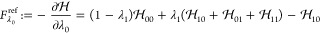
28
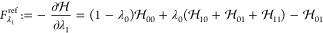
29for the system with four forms. Subsequently, the relative deviations (*F*_λ_ – *F*_λ_^ref^)/*F*_λ_^ref^ were determined at different multipole orders *p* and tree depths *d*. Note that the reference and MAHI forces were calculated at the same multipole order *p*. This exactly quantifies the MAHI forces deviations from those obtained with naively computed HI, while avoiding the force differences emerging due to different precision levels. The λ values were kept constant at randomly chosen λ = 0.345 for the system with two forms and at λ_0_ = 0.345 and λ_1_ = 0.721 for the system with four forms.

#### 3.1.2 Random Systems with Varying Number of Sites and Forms

To quantify the scaling behavior of our MAHI scheme with respect to the number of sites and the number of forms per site, we generated random systems with varying numbers of particles (250–12,725,399), sites (1–512), and forms per site (2–16).

#### 3.1.3 Random System with Varying Number of Particles

To quantify how the computational effort of MAHI scales with the total number of charges in a system, we considered a series of random position systems with particle numbers ranging from 250 to 33,554,432. A typical fraction of one 10-atom titratable site per 4000 atoms was chosen in each case, estimated from a solvated globular lysozyme protein.^50^

#### 3.1.4 Benzene Ring in Water

**Figure 4:**
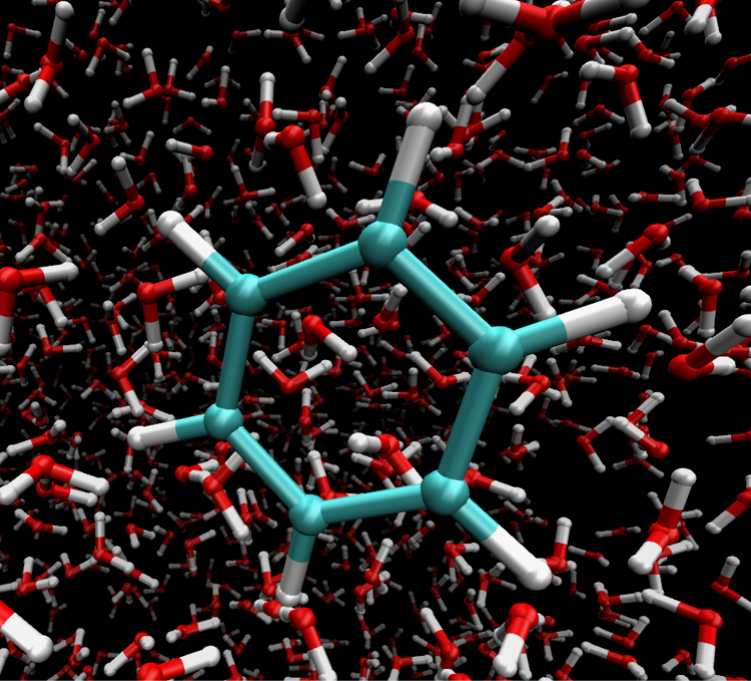
Benzene ring solvated in water. The ball-and-stick drawing shows hydrogen atoms in white, carbon atoms in cyan, and oxygen atoms in red.

To test the overall accuracy of the λ forces provided by MAHI within GROMACS, we considered a solvated benzene molecule, comprising a C_6_H_6_ ring and 2,161 TIP3P water molecules^51^ in a 4 × 4 × 4 nm^3^ box using the Amber99sb*-ILDN force field,^52,53^ as shown in Figure 4. The reference  values were calculated for λ covering the range between zero and one, where the benzene molecule carries its full charge at *t* = 0.0 ps (λ = 0), while it is completely uncharged at *t* = 2.0 ps (λ = 1). All the reference values were obtained by GROMACS thermodynamic integration (TI) using PME electrostatics with fourth order B-spline interpolation, 0.12 nm grid spacing, and 1.1 nm cutoffs. For the FMM test runs, *p* = 8 and *d* = 3 were used. For all simulations, a 2 fs time step was used while constraining the bonds of the water molecules with the SETTLE algorithm,^54^ and all other bonds with LINCS.^55^

#### 3.1.5 Constant pH Simulation Systems in GROMACS

To assess the computational overhead of the entire constant pH GROMACS implementation relative to fixed charge FMM simulations, several simulation systems containing a protein with one or more titratable sites solvated in TIP3P^56^ water and Na^+^ and Cl^–^ ions (150 mM) were considered. These tests used the CHARMM36m force field,^56^ a 2 fs time step and a 1.2 nm van der Waals interaction cutoff.

The simplest systems contained a single solvated titratable glutamic acid (Glu) residue within cubic boxes of edge lengths 5, 6, 7, and 8 nm, comprising in a total of 12,125, 20,996, 33,552, and 50,682 atoms, respectively. To assess how the addition of titratable sites affects overall GROMACS performance, solvated hen egg lysozyme (PDB code 2LZT)^50^ and staphylococcal nuclease (SNase) mutant ΔPHS (PDB code 3BDC)^57^ with different numbers of titratable sites were benchmarked. Both proteins contain numerous histidine (His), aspartic (Asp) and glutamic acid (Glu) residues. The lysozyme systems contain 1–10 protonatable residues in a 6.5 × 6.5 × 6.5 nm^3^ box, totaling 26,761–26,779 atoms. The SNase system contains 50,749–50,760 atoms in an 8.0 × 8.0 × 8.0 nm^3^ box with 1–20 protonatable residues.

#### 3.1.6 Hamiltonian Interpolation vs Charge Interpolation

To characterize the differences between HI and QI for typical simulation systems, the relations derived in Section 2.4 were verified numerically. For this purpose, the FMM code was modified to perform either HI or QI λ-dynamics, while simultaneously reporting both  and .

The effect of this difference on protonation/deprotonation kinetics was assessed by counting the number of transitions between protonation states during simulations of equal lengths. To this end, a single methyl-blocked Glu residue solvated in water was used as a test system. For the sake of simplicity, a two-state model without tautomery was used, and only the Glu residue was made protonatable.

First, ten replicas of the Glu system were simulated for 30 ns using the CHARMM36m force field^56^ at pH 4.4 (the p*K*_a_ of Glu) with both QI and HI. The dynamic barrier optimization was disabled, and the double well barrier thus set at an unchanging height of 5 kJ/mol. The double-well potential is illustrated in Figure 1 of our companion paper,^34^ with the barrier height defined in its Supporting Information. To ensure unbiased comparison of transition rates, the *V*_MM_(λ) potential was calibrated for a flat energy landscape at pH = p*K*_a_ in both HI and QI as well. Details on the *V*_MM_ potential and the calibration process are in our companion publication.^34^ As a typical protein system, SNase was simulated with the same protocol and conditions as the single residue, using 40 replicas of 60 ns each.

### 3.2 Benchmarking Procedure

All benchmarks were run on a compute node with an NVIDIA GeForce RTX 4090 GPU and an AMD Ryzen Threadripper 1950X 16-core processor with 32 GB of RAM running Scientific Linux 7.9. GROMACS with FMM was compiled using GCC 9.4.0 and CUDA 12.2, thread-MPI, and AVX2_256 SIMD instructions, and with OpenMP and hwloc 2.1.0 support. The benchmarks with GROMACS used one thread-MPI rank and 16 OpenMP threads and were run for several thousand time steps. Because memory allocation and load balancing typically slow down the first few hundred time steps, timings were collected only for the second half of each run. All reported performances are averages over three runs. Each of the FMM standalone benchmarks was averaged over several to several thousand runs, depending on tree depths and particle counts.

## 4 Results and Discussion

### 4.1 Accuracy

In order to test the accuracy of the proposed scheme, the forces  obtained with corrected FMM were compared with reference solutions (eqs 27 and 28) generated by direct (redundant) computations of the separate Hamiltonians. Additionally, FMM-derived forces  were compared to those from a standard GROMACS TI simulation using two PME calls (see Section 3.1).

#### 4.1.1 Accuracy of the Random System with Two Forms and Four Forms

**Figure 5:**
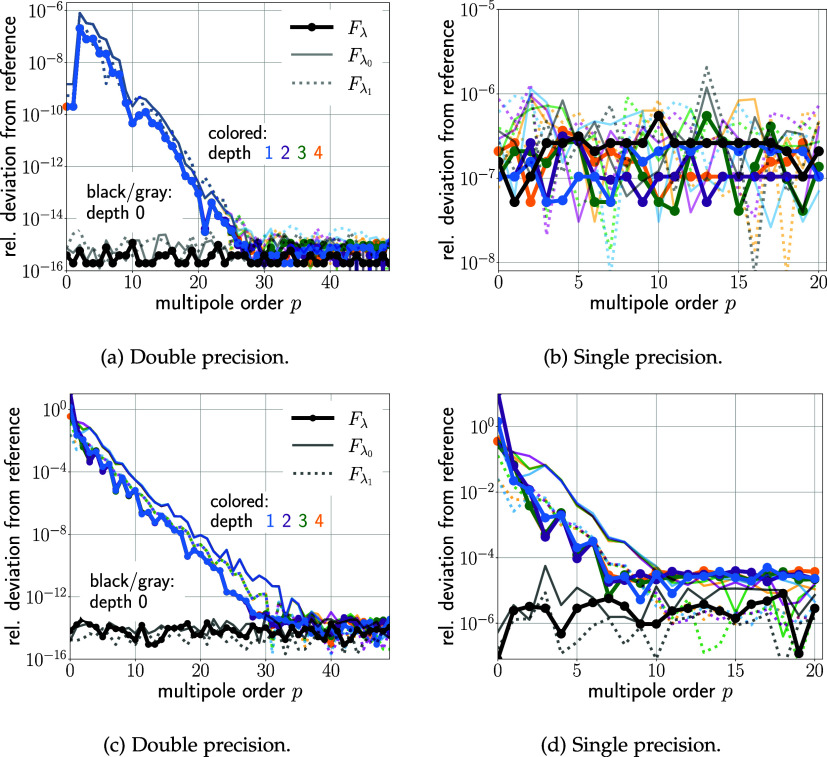
Quantification of the accuracy of the MAHI scheme. The plots show the relative deviation of the MAHI forces from the reference forces for the 1010 particle test system with typical particle distribution (a) and (b), and with hypothetical worst-case particle distribution (c) and (d). FMM depth *d* indicated by color, *d* = 0 (black), *d* = 1 (blue), *d* = 2 (purple), *d* = 3 (orange).

Figure 5 shows the relative deviations of forces *F*_λ_ from the reference forces for two forms and *F*_λ_0__, and *F*_λ_1__ for four forms. First the case of tree depth *d* = 0 was considered. Here, all correction terms are evaluated directly, hence MAHI is expected to be identical to the reference forces within numerical precision. Indeed, the relative deviations for *d* = 0 (depicted by the black and gray curves) are within numerical precision for both double (a) and single precision (b) calculations for all considered multipole orders.

In contrast, for larger tree depths *d*, the colored curves in Figure 5 show differences to the reference forces particularly for low multipole orders *p*, which decrease as expected with increasing order. The largest relative deviations (up to 10^–6^) are seen for *p* ≤ 3. For *p* ≥ 28, the relative deviation reaches numerical (double) precision, indicating that the scheme does not introduce additional approximation errors.

Interestingly, the maximum relative deviation for double precision is about 10^–6^, which is approximately single precision accuracy level. Thus, one would expect no significant deviations and no dependence on multipole order when tested at single precision. In fact, as can be seen in Figure 5b, the deviation remains at the same level over the entire range of *p*.

Next, the relative deviations of the forces on the λ particle for the worst-case distribution of particles was quantified. Figure 5 shows a qualitatively similar behavior of the relative deviations in double precision compared to the more realistic case above. In particular, at tree depth *d* = 0 the deviations remain at numerical (double) precision, whereas at larger depths increasing accuracy for increasing multipole order *p* is achieved. In contrast to the typical case, however, overall much larger deviations are seen, as expected for the worst-case particle distribution.

The observed convergence with increasing multipole order confirms that the deviations are due to the truncation of the multipole interactions. The larger deviations compared to the typical case can be attributed to the intended unfavorable nonclustered site particle distribution, which implies that essentially none of the mutual interactions are calculated directly in the precalculated charge-scaled Hamiltonian. As a result, all corrected interactions may have large deviations. This is even more pronounced in single precision, where the relative deviations stay close to numerical (single) precision at tree depth *d* = 0. However, for larger depths *d*, the relative deviations do not fall below 10^–5^. We anticipate that this is due to large errors in multipole expansions built for only a few particles, and due to numerical cancellations that occur when summing of larger potential values with very small corrections.

Similar overall accuracies are seen for the whole λ range (data not shown).

#### Comparison of  for FMM vs PME

4.1.2

**Figure 6:**
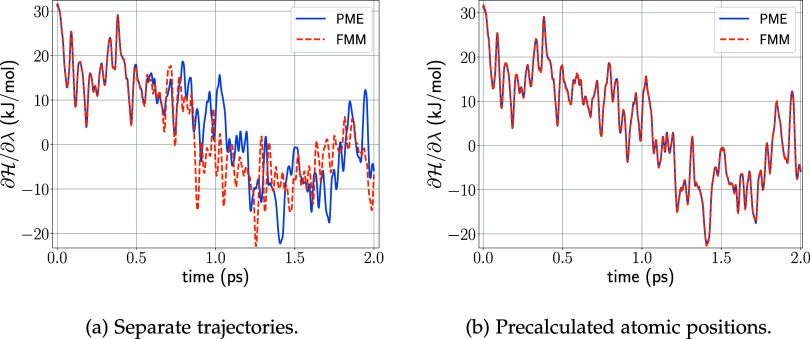
Comparison of the force acting on the λ particle as calculated by PME and FMM for uncharging benzene. The benzene ring carries its full charge at *t* = 0.0 ps (λ = 0), while at *t* = 2.0 ps (λ = 1) it is fully uncharged. FMM-computed  values in orange, PME in blue.

We next compared our FMM implementation to PME electrostatics using the benzene ring solvated in water. To this end, we used single precision FMM with multipole order *p* = 8 and depth *d* = 3. Figure 6a shows  along a 2 ps trajectory calculated using PME (blue) and our FMM scheme (orange dashed), respectively. During this simulation, λ covered the full range between zero and one. As expected, essentially identical λ trajectories are seen for the first several hundred integration steps; thereafter initially minute deviations amplify due to the chaotic nature of the dynamics of strongly interacting multiparticle systems. However, using exactly the same precomputed and stored input atomic positions for both PME and FMM, matching derivatives (Figure 6b) were obtained over the entire λ range. The root-mean-square error between FMM and PME forces is approximately 0.22 kJ/mol.

### 4.2 Performance

Next, we assessed the computational performance of MAHI. To this end, we first characterized the scaling behavior with the number of sites, forms, and particles; second, we evaluated the overall performance of GROMACS with corrected FMM is also evaluated in the context of constant pH λ-dynamics simulations.

#### 4.2.1 Scaling with Increasing Number of Sites

**Figure 7:**
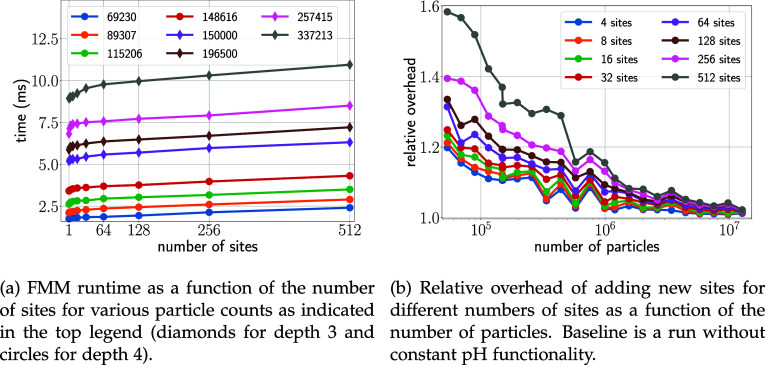
Scaling of the FMM based MAHI scheme for different numbers of sites and particles. Each site contains two forms. Results are shown only for the optimal tree depth.

As discussed in Section 2.3, we expect MAHI to scale linearly with the number of sites. Figure 7a shows the average runtime for increasing the number of sites and for various particle counts between 70k and 340k at depths *d* three and four, chosen for optimal performance. For larger number of sites, the linear increase can clearly be seen, whereas for smaller number of sites a steeper increase is seen, due to constant costs associated with incorporating additional data structures and functions for correction calculations. Moreover, for a small number of sites, constant pH related kernels do not achieve their optimal performance due to the insufficient computational load required to optimally utilize the underlying hardware. This effect can be seen more clearly in Figure 7b, which shows how the additional effort of the constant pH functionality scales with the number of particles for a given number of sites (different colors). As can be seen, the relative performance overhead is quite small, and for realistic systems with moderate numbers of sites generally below 20%. For larger systems of several million particles the overhead becomes negligible.

#### 4.2.2 Scaling with Number of Forms

**Figure 8:**
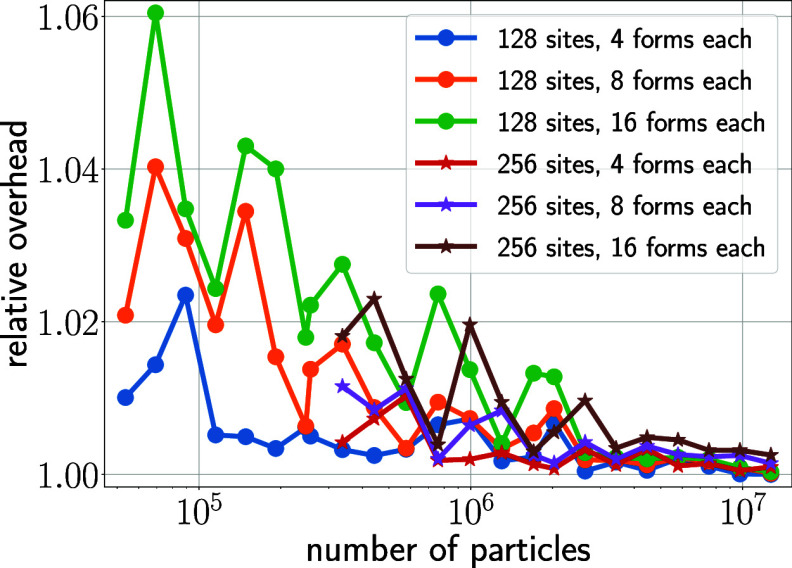
Costs of adding new forms to existing sites. Results are shown for FMM tree depths of *d* = 3 (circles) and *d* = 4 (stars) for the random systems. Baseline is the FMM performance with sites containing two forms (one λ value).

Next, we studied how adding new forms to existing sites impacts performance. Because additional forms require separate recalculations (see Figure 12), one expects a moderate linear increase of the additional effort. Figure 8 quantifies the resulting overhead for selected numbers of sites and forms (different colors). Notably, adding new forms does not markedly affect performance, with an additional overhead of generally below 5% and below 2% for larger systems. Here, the overhead is entirely due to the increase of calculations performed by the kernels. No additional data structures or kernel calls are required, which explains the small overhead.

#### 4.2.3 Scaling with Number of Particles

**Figure 9:**
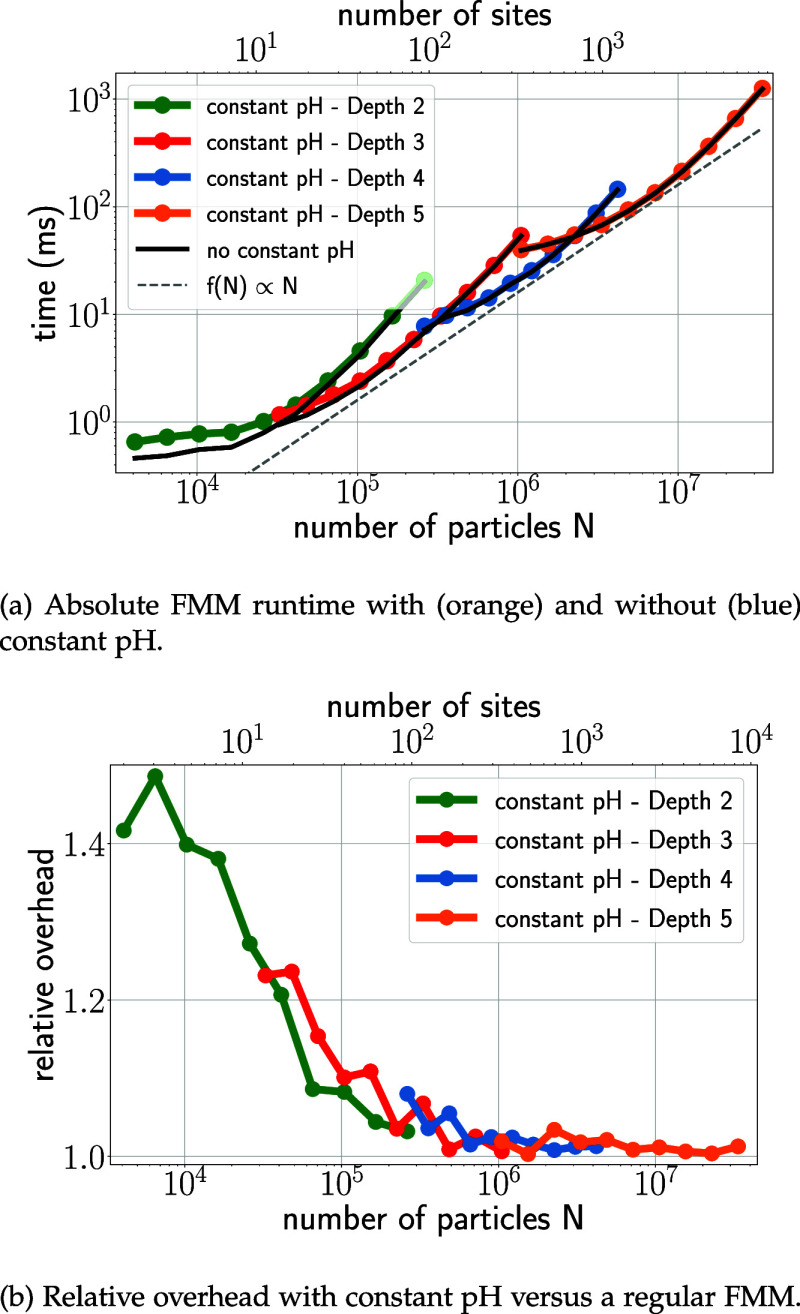
Comparison of FMM runtime with and without constant pH. This benchmark uses one site with ten particles for every 4000 particles in the system, as estimated from lysozyme.

To characterize the scaling of the computational effort of MAHI with the number of particles under realistic simulation conditions, Figure 9a shows the absolute runtimes of FMM with and without constant pH functionality. The characteristic behavior of FMM is evident in both cases, with piecewise quadratic scaling for different choices of tree depth *d*. A proper choice of *d* results in an overall linear scaling (dashed line) with system size. Notably, the scaling of FMM is nearly unaffected by the constant pH overhead, with small runtime differences seen only for systems with fewer than 10^5^ particles. This finding is also reflected in the relative overhead of including constant pH (Figure 9b), which decreases from approximately 50% for very small systems (below 10^4^ particles) to below 10% for typical system sizes, and to nearly zero for large systems. Overall, the addition of the constant pH feature has minimal impact on FMM scaling.

#### 4.2.4 GROMACS Performance with FMM and Constant pH

**Figure 10:**
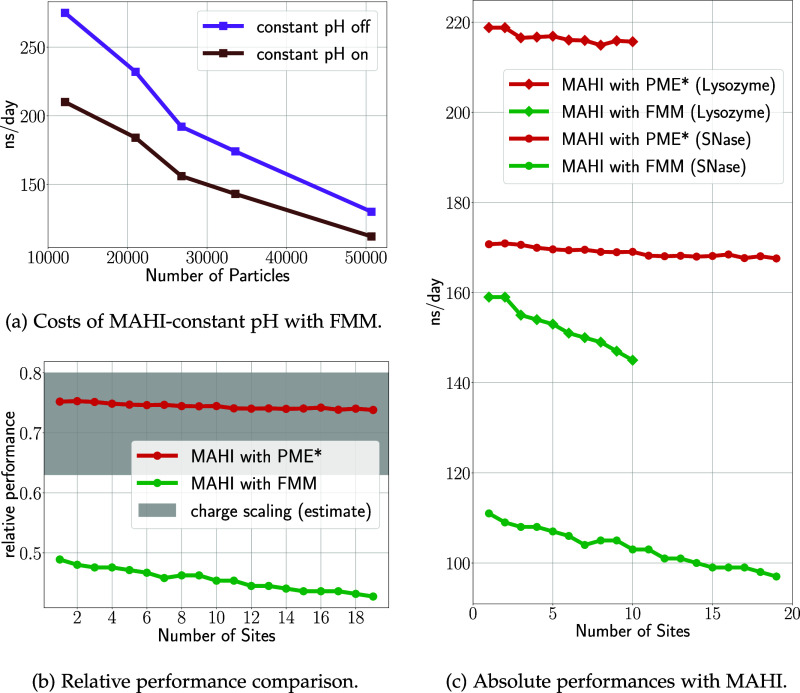
GROMACS performance with FMM and PME electrostatics for different constant pH simulation systems. (a) A single titratable Glu in water–water boxes of increasing size. (b) Relative performance for SNase with PME- and FMM-based MAHI compared to a fixed protonation simulation. Costs of PME-based charge scaling estimated from Aho et al.^11^ (c) GROMACS performance with varying number of sites for FMM- and PME-based MAHI. *Preliminary results.

The previous benchmarks assessed the performance of the constant pH FMM as a stand-alone solver. Our final performance test, therefore, addressed the total runtime of a constant pH FMM GROMACS simulation, which also involves calculating the dynamics of the λ particles. This is likely the most relevant benchmark for most users. Figure 10 compares the constant pH performance to a regular GROMACS FMM run for a single protonatable residue in a box of increasing size (a), and for lysozyme and SNase (c). Here, too, a constant pH overhead of about 25% is seen for small systems of about 10,000 particles, dropping below 10% for systems with ≈50,000 particles. Additionally, the costs for adding new titratable sites range from 0.5 to 1 ns/day per site, as shown for small solvated proteins in Figure 10c. Thus, for typical numbers of titratable sites in biomolecular systems the overhead due to the number of sites is negligible in both absolute and relative terms. Overall, the computational effort of MAHI is essentially independent of the number of sites, with only a minor impact (typically below 10%) when increasing the number of sites.

Since the corrections  are applied to the charge-scaled potential  precalculated from a charge-interpolated system , it is actually irrelevant what method is used to obtain  as long as it is sufficiently accurate. This finding opens up new routes for further performance improvements by using faster methods to obtain . Along this lines, we tested MAHI with PME such that HI-based λ forces can be obtained.

Indeed, this hybrid approach proves advantageous in terms of overall performance. Figure 10c quantifies its performance, using the same test systems and parameters as above for the FMM-based MAHI. We observed a 40 and 55% performance improvement for the lysozyme and SNase system, respectively.

Additionally, Figure 10b compares the performance of MAHI to the charge-scaling method in terms of relative overhead. The performance of a charge scaling simulation (gray area in the panel) is in the range of 0.63–0.8 times the performance without constant pH, as estimated from Figure 6A,B in Aho et al.^11^ PME-based MAHI (red curve) will also be in this range based on our performance estimates. However, additional testing is required to confirm the accuracy of PME-based MAHI for constant pH simulations.

#### 4.2.5 Differences between Hamiltonian and Charge Interpolation

Having discussed the mathematical differences between HI and QI in Section 2.4, we will now assess the practical implications of these differences in constant pH simulations.

For a quantitative comparison of HI and QI, both constant pH setups must be equally well calibrated. To this aim, the Glu reference compound in water at pH = p*K*_a_ was simulated for both HI and QI separately, using the acquisition protocol described in our companion publication.^34^ In both simulations, the ratio of time spent in the protonated and deprotonated states, as well as the average λ value, was 0.50 ± 0.02, allowing for a rigorous comparison.

**Figure 11:**
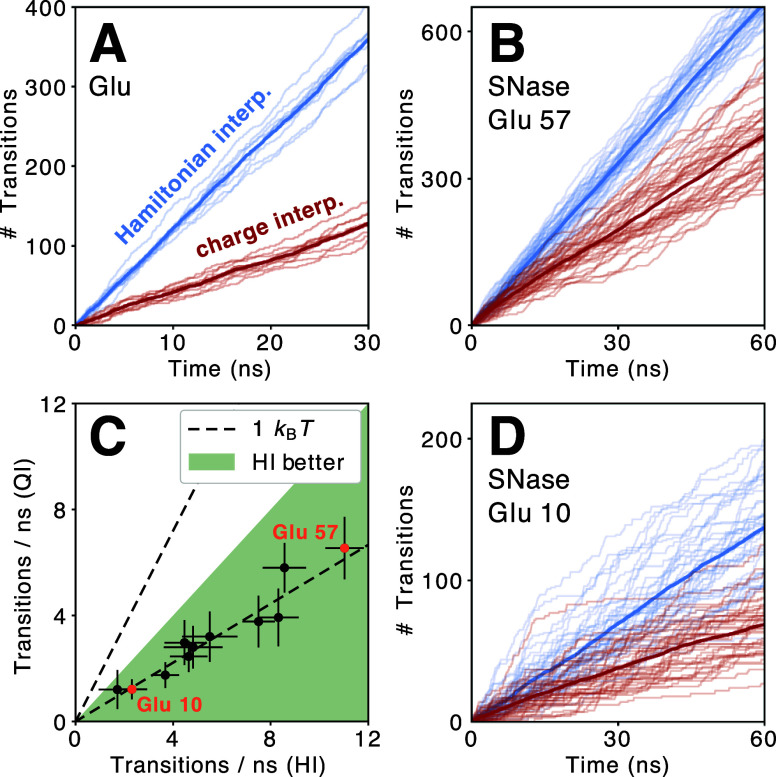
Comparison of Hamiltonian interpolation (HI) and charge interpolation (QI) constant pH simulations. Cumulative number of transitions for a single Glu residue in water (A), and for Glu 57 (B) and Glu 10 (D) in the SNase protein. Transparent lines correspond to individual replicas, solid lines to the average; HI in blue and QI in red. In (C), the transition rates of all Glu residues in SNase are compared. Error bars give standard deviation across replicas. Dashed line indicates how much an additional barrier of 1 *k*_B_*T* reduces the transition rate in a TST model.

We started by investigating the differences for the single Glu residue in water. Figure 11A shows the cumulative number of transitions over time between protonated and deprotonated states for HI and QI. With about 12 transitions per nanosecond for HI versus only four for QI, the transition rates are very different. Using a Transition State Theory (TST) model,^58,59^ this can be translated into an additional barrier of about 1 *k*_B_*T* for QI. This barrier corresponds to the harmonic potential identified in Section 2.4.

We then investigated the relevance of this additional barrier for larger proteins, using the SNase system. In the protein environment, transition rates vary for each residue due to the different local environments. For instance, Glu 57 has the highest transition rate for both HI and QI (Figure 11B,C), with a total of over 600 and 300 transitions, respectively, whereas Glu 10 shows less than 300 transitions in 60 ns (Figure 11D). While the transition rate for a given residue varies between replicas and over time, the average rate (Figure 11C) is consistently higher for HI than for QI. In the TST model, this difference corresponds to an additional barrier of about 1 *k*_B_*T* (black dashed line) as for the single Glu residue in water.

**Figure 12:**
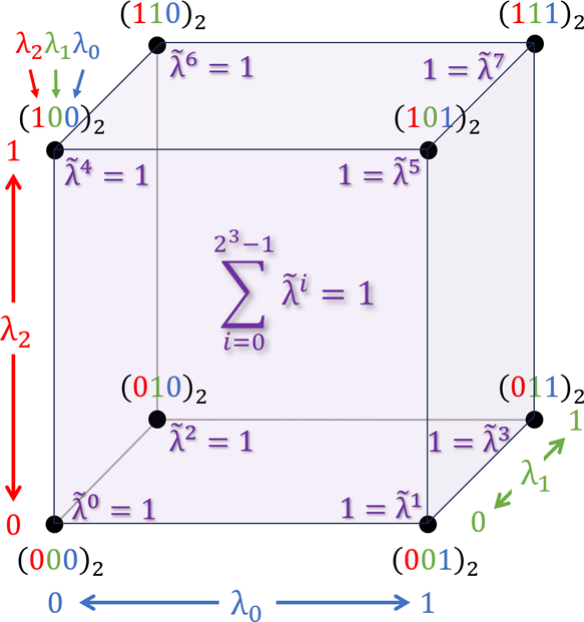
Sketch showing how λ is mapped to λ̃. Multistate model for an exemplary site with eight forms, demonstrating the mapping of λ values to their corresponding λ̃ values.

We therefore conclude that in practical simulations, in the absence of other variable factors such as an automatic barrier optimization,^34^ HI leads to higher transition rates than QI. However, the magnitude of this effect, which is related to *k*(**r**) (see eq 26), is a function of the parametrization of the residue of interest (charge, bond length) and can therefore vary.

## 5 Conclusions

Here, we derived and evaluated a constant pH λ-dynamics extension of our GPU-based FMM that implements rigorous Hamiltonian Interpolation (HI). It provides an efficient and scalable multipole-based computation of the difference between HI and precomputed charge-scaled Hamiltonians. This implementation avoids redundant electrostatic calculations that typically arise when calculating interactions between particles not belonging to any site, e.g., environment, in systems with many protonatable sites. In addition, we demonstrate the integration of MAHI into the FMM framework and into the GROMACS software suite, enabling efficient and straightforward constant pH MD simulations.^34^

We assessed the accuracy of the extension by comparing the forces acting on λ particles to reference forces, and found that these forces are within the accuracy range of the tested multipole order *p*, for both single and double precision. In particular, the forces obtained are within numerical accuracy of those obtained by the GROMACS free energy module.

Benchmarks of MAHI showed that for biomolecular applications, and particularly for moderate to large MD systems, the inclusion of typical numbers of titratable sites does not markedly affect the simulation performance, and that the involved computational overhead scales linearly with the number of sites and forms. Benchmarks of the entire constant pH GROMACS implementation showed similar performance, demonstrating that pre- and posthandling of the data does not introduce any performance bottleneck. Overall, for a system comprising 100,000 particles, the overhead is less than 10% compared to runs without constant pH.

To explore further ways to increase performance, we tentatively combined MAHI with PME. In particular, we computed the charge-scaled Hamiltonian with PME, and then added the HI-QI difference with MAHI. This exciting approach requires further testing and benchmarking, but promises another 40% performance improvement. In addition to this practical benefit, this test demonstrates the flexibility of MAHI and shows that it can be combined with other electrostatic solvers such as PME, which was previously deemed impractical.^11^ Initial tests indicate that PME-based MAHI simulations will not be much slower than charge scaling simulations.

Closer analysis of HI and QI revealed differences between the two interpolation schemes. We demonstrated that QI introduces protonation/deprotonation free energy barriers that are generally higher than those for HI. This reduces sampling efficiency and hinders the convergence of, e.g., p*K*_a_ calculations. To mitigate this issue, we developed and evaluated an automatic barrier optimization protocol, described in our companion publication.^34^

In addition to MAHI, FMM enables constant pH simulations of systems with open boundaries without further modifications. The combination of open boundaries and nontruncated treatment of long-range interactions is unique among fast electrostatics solvers currently used for MD. FMM performs very well in this area, for example for droplet systems used in the simulation of mass spectrometry experiments.^41^

As a next step, we aim to exploit the scaling properties of the FMM to enable constant pH for larger systems using multi-GPU, multinode parallelism, in line with the trend toward exascale computing. We anticipate that our constant pH approach will also scale favorably on exascale machines, as it is composed primarily of independent tasks that can be divided and parallelized with minimal communication overhead. In particular, we expect that the parallelization of FMM avoids the considerable communication bottleneck and unfavorable scaling of the FFT required for PME. Finally, MAHI is directly applicable not only to constant pH, but also more generally to all free energy computations that rely on the calculation of .

A GROMACS version with the FMM-based constant pH module is available for download at https://www.mpinat.mpg.de/grubmueller/gromacs-fmm-constantph.

## Figures and Tables

**Table 1:**
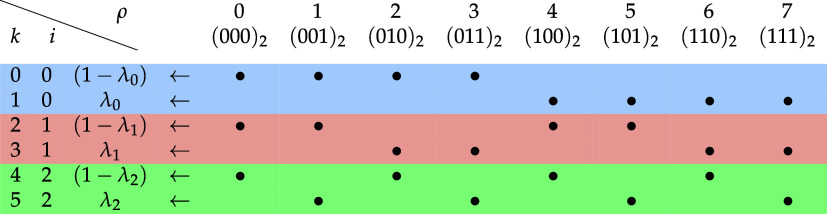
Mapping of the MAHI Correction Terms^a^

a Translation of the correction terms associated with the λ̃ values back to the terms for corrections of the forces on the initial λ values, here for an exemplary site with eight forms (enumerated 0–7). The dots indicate which term applies to which original λ force.

## Appendix

In the following, we present the mathematical foundations that lead to the general formulation (eq 6) presented in this work.

As stated in eq 4, the Hamiltonian

describes a system consisting of two sites with two forms each. It is composed of four sub-Hamiltonians , , , and

These sub-Hamiltonians describe the system in all four different protonation combinations of the two sites with two forms; both sites protonated (00), one of two sites protonated (01 and 10), and both sites deprotonated (11).

First, consider the interactions between the λ independent environment particles (printed black in the above equations)

Since (1 – λ_1_)(1 – λ_0_) + (1 – λ_1_)λ_0_ + λ_1_(1 – λ_0_) + λ_1_λ_0_ = 1 and since the charges *q*_*i*_, *q*_*j*_ are independent of λ, the sums reduce to

Next, consider the interactions between the site particles and environment particles (blue terms in the above equations)

For example, interactions between site 0 and environment are

Since (1 – λ_1_) + λ_1_ = 1 the higher λ terms cancel out and the above expression reduces to

Further, as the charges of the environment are independent of λ_0_, the λ_0_ terms can be put directly into the sums leading to charge-scaled interactions between site 0 and environment

The same holds for site 1, hence all interactions between the sites and environment reduce to

which are interactions between scaled charges and the environment.

Considering the interactions between particles of site 0 and of site 1 (site–site interactions, green color in the equations above), similarly to site–environment interactions, it holds

This is valid for the same reason as in case of the site–environment interactions; for site 0 the charges *q*^0,0^ and *q*^0,1^ are λ_1_ independent and for site 1 the charges *q*^1,0^ and *q*^1,1^ are λ_0_ independent.

When considering interactions between particles belonging to the same form of the same site (red terms in the above equations), all interacting charges depend λ_0_ values. Again, consider only site 0 for clarity.

As in previous example, the λ_1_ cancels out leading to

Here, in contrast to the previous three parts, the entire interactions are scaled and the sums do not reduce to interactions between charge-scaled particles.

Now, consider a system with one site and four forms according to eq 33

Here, as in the previous examples, the partial Hamiltonians  and  reduce to interactions between scaled particles. The complete Hamiltonian reads

where only intra-site interactions are weighted with the λ̃ values obtained as shown in eq 34.

The differences in intra-site interactions are the foundations for MAHI derivation, which is constructed as follows. For each site σ = 1,...,*M* we construct a list

30

of length *L*^(σ)^ := ⌈log_2_(*#S*^(σ)^)⌉ containing pairs (1 – λ, λ) connecting distinct forms of the site *S*^(σ)^, as in the multi-state model. Calculating the Cartesian product

31

yields *#S*^(σ)^ lists of length *L*^(σ)^. Each Ω̃_ρ_^(σ)^ is a unique product of the 1 – λ and λ values, resembling the binary representation of the λ combinations depicted in Figure 12, where 1 – λ := 1 and λ := 0 and the index of the λ pair in eq 30 represents the bit position. Taking the products of all elements of each Ω̃_ρ_^(σ)^

32

yields λ̃ values used in the general formulation. With this, the entire Hamiltonian, consisting of an arbitrary number of states  with an arbitrary number of forms per site , can be transformed to into an equivalent general formulation where the λ’s are no longer factors in front of the sub-Hamiltonians, but weigh the different forms of a site.

The construction of multiple forms proceeds as follows. Let us consider a system with one site (we omit the site index σ) and four forms

33

with prime notation to emphasize the difference to eq 4. Here, in contrast eq 4, the sub-Hamiltonians , where , represent four different protonation forms of the same site. Multiplying all λ terms that belong to the same site yields

34

The index ρ of each λ̃^(σ,ρ)^ is a decimal representation of each binary element of . Figure 12 depicts the enumeration of different site-forms and the corresponding λ and λ̃ values for eight forms.

In practice, we encounter Hamiltonians  for multiple sites σ = 1, ..., *M* that consist of sub-Hamiltonians  and . The straightforward multiplication of all λ values (from different sites) to obtain λ̃^(σ,ρ)^ for each site *S*^(σ)^ according to eq 34 is still valid because the site–site interactions are calculated between charge-scaled particles, thus, the site independent λ values cancel out.

To calculate the forces acting on the original λ particles, the scaled correction terms  are subtracted from the corresponding energies derived from the interactions between particles of the same site-form *S*^(σ,ρ)^ only. To determine the derivatives , the indices ρ of each site σ are mapped back to the indices *i* = 0, ..., *L*^(σ)^ – 1. Table 1 shows the scheme for back-mapping eight corrections with indices ρ = 0, ..., 7 to three λ values with indices *i* = 0, 1, 2.

This is achieved by continuously enumerating indices of λ-pairs created according to eq 30

35

and by taking the Cartesian product of these indices

36

This yields the index tuples of the original λ values. This procedure follows the same logic as in eq 31 but operates on the indices to track which λ values need to be excluded from the derivative calculations as the calculation of the derivatives with respect to the original λ values from the corrections

37

excludes the contribution of the λ value itself. With this, we calculate intermediate correction terms (see Table 1)

38

where  is the potential of the charge-scaled system defined in eq 12. The restriction  ensures that only the relevant terms contribute to the final λ force as shown in Table 1. Finally, the forces on the λ particles are obtained with

39

In the following, we derive the expression for the correction charges  as given in eq 14. The potential of the charge-scaled system is given by

40

First, we consider the box-box interaction part  (see eq 13) of the entire correction . Let us denote the correction potential with . The correction terms are evaluated only on the part of the particles *N*^σ^ belonging to the site *S*^(σ)^. Hence, for some fixed site-form *S*^(σ,ρ)^ we correct the charge-scaled Hamiltonian with

41

Here, we have only retrieved the env-site and intra-site parts of the energies. These, however, are the only parts required for the calculation of the energy differences, i.e. the forces on the λ particles. Note that the env-site term does not include a factor of 1/2 because it involves an iteration over non-overlapping set of particles. For the case of two forms (ρ and ρ′) the forces on the λ particles are given by

Next, we describe the use of the lattice correction part . The lattice operator  approximates the periodic contribution from the infinite replicas of the simulation box surrounding the central box. It computes the periodic system energy using the multipole-to-local transformation,^43^ applied to the multipole expansion of the central cell ω with

42

where *p* is the multipole order, which controls the accuracy of the approximation and μ is the local moment calculated by the lattice operator. A detailed derivation of the lattice operator can be found elsewhere.^48^ Similar to the derivation of  (eq 41) for the periodic part of the energy we get

43

where ω̊(**x**_*i*_) represents a chargeless multipole expanded with particles at positions **x**_*i*_, μ̃ is the precalculated charge-scaled local expansion, and μ̃^(σ,ρ)^ is the charge-scaled local expansion expanded only for the *N*^(σ)^ particles within the specified site-form *S*^(σ,ρ)^. Further rearranging yields

44

which are the energy terms needed for the calculation of the λ forces.

Finally, we explain the dipole compensation correction term , presented in eq 22, as

Using the definitions ω̂^(σ,ρ)^ := ω(**Q̂**^(σ,ρ)^), ω̃ := ω(**q̃**), , and  we rearrange the terms and obtain

45

In contrast to the corrections  and , correcting the dipole compensation energy requires accounting for the site-env (energy on environment particles due to interactions with site particles) and env-site (energy on site particles due to interactions with environment particles) terms, as they differ and are necessary to obtain the correct λ forces.
